# Retraction Note: Simulation and spatiotemporal pattern of air temperature and precipitation in Eastern Central Asia using RegCM

**DOI:** 10.1038/s41598-019-49154-5

**Published:** 2019-09-18

**Authors:** Xianyong Meng, Aihua Long, Yiping Wu, Gang Yin, Hao Wang, Xiaonan Ji

**Affiliations:** 1 State Key Laboratory of Simulation and Regulation of Water Cycle in River Basin & China Institute of Water Resources and Hydropower Research, Beijing, 100038 P. R. China; 2 Department of Earth & Environmental Science, Xi’an Jiaotong University, Xi’an, Shaanxi Province 710049 P. R. China; 3 Xinjiang Institute of Ecology and Geography, Chinese Academy of Sciences, Urumqi, 830011 P. R. China

Retraction of: *Scientific Reports* 10.1038/s41598-018-21997-4, published online 26 February 2018

The authors are retracting this Article.

In this study, we used RegCM to downscale the ERA40 and NCEP climate data in the Xinjiang area, and then compared the simulated data with extrapolated data from CRU.

Extrapolated data were used because using observational data would have led to poor performance of the model, RegCM. As a result, conclusions reported in the paper – increasing precipitation in South Tianshan, and a decreasing trend in the north – are unsupported, and are misleading to the research community and the public. Therefore, we are retracting this article. We apologize for the inconvenience caused.

All authors agree with the retraction.

